# Field investigation of bicycles for indirect bridge structural health monitoring

**DOI:** 10.1007/s13349-024-00885-8

**Published:** 2024-12-21

**Authors:** Richard May, Hwa Kian Chai, Thomas Reynolds, Yong Lu

**Affiliations:** Institute for Infrastructure and Environment, The University of Edinburgh, Thomas Bayes Road, Edinburgh, EH9 3FG Scotland

**Keywords:** Crowdsourcing, Bicycle, Bridge, Indirect bridge monitoring, Human–structure interaction, Environmental and operational variation

## Abstract

Indirect structural health monitoring (iSHM) for bridges typically utilises motorised vehicles. A large number of pedestrian and cycle bridges worldwide cannot practically be accessed by these vehicles. Nevertheless, such bridges are equally susceptible to ongoing accumulation of defects. This paper reports field investigation of using bicycles as exciters and sensor carriers for identifying bridge modal parameters. Data are gathered simultaneously from the moving bicycle and the subject bridge to reduce ambiguity. Bridge modal frequencies estimated using bicycle-mounted sensors are compared to baseline properties estimated using ambient and pedestrian heel drop inputs. Changes in baseline modal frequencies are observed to be correlated with varying temperature, a known cause of environmental and operational variation (EOV). The possible pollution of recorded signals due to human–bicycle interaction dynamics is considered. The combined rider–bicycle–bridge system is observed to exhibit nonstationary frequency behaviour during freewheeling traversals, and bridge resonance due to harmonic pedalling forces is demonstrated. Increased pedalling cadence is correlated with reduced frequency nonstationarity for the combined system. It is suggested that this could be due to an increase in the rider–bike subsystem fundamental frequency caused by rider posture. Collectively, these observations suggest the potential for the use of fleets of bicycles for iSHM, while highlighting the need for greater understanding of potential confounding due to rider–bicycle and rider–bicycle–bridge interaction dynamics as a source of EOV.

## Introduction

Bridges form a part of the critical infrastructure upon which society relies. Interruptions to service have significant impact [1]. However, all materials contain defects [2] and these may eventually lead to an interruption of the continued safe use of the bridge if not identified, quantified and appropriately mitigated in a timely manner. Structural health monitoring (SHM) aims to supplement or perhaps replace the traditional model of scheduled visual bridge inspections with continuous data gathering. The collected data are evaluated in accordance with the accepted procedure (first proposed by Rytter [3]) of damage identification, localisation, quantification and assessment of consequences.

Bridge damage can be conceptualised as a reduction in strength or stiffness usually associated with undesired changes in mass, material integrity or damping. Changes to modal parameters (such as frequencies, damping ratios and mode shapes) have been pursued as indicators of damage [4, 5]. The use of such signal features for damage detection is in part motivated by perceived limitations to visual inspection techniques. Although such inspections are an established part of bridge management, some are concerned that damage may not be visually apparent [6], and that the inspection process is labour-intensive [7] and subjective [4]. Changes in bridge modal parameters arguably only have meaning relative to a known ground truth initial state, since all damage detection relies on comparison of two structural states [2]. Thus, structural system identification (estimating the modal parameters) at an early stage post-construction is valuable alongside ongoing monitoring.

Confounding is introduced by the class of effects collectively termed environmental and operational variation (EOV)—these can cause changes to estimated modal parameters [8], potentially obscuring or preventing damage visibility, or creating false positives. Mitigation of EOV is typically implicit or explicit [9]. The implicit approach includes approaches such as splitting recorded data into segments within which the EOV-induced effects are minimal [10], or processing the data to extract features which are EOV-insensitive (for example, Cross et al.’s [11] study which included such techniques as principal component analysis). The explicit approach involves directly modelling the relationship between EOV and the structural response (for example, the regression approach employed by Peeters and De Roeck [12]). In both cases, it is beneficial to undertake continuous monitoring of both the structure and its environment, thus establishing baseline expected behaviour. The resulting data set should encompass the full scope of expected operational conditions [10].

The benefits of early and continuous monitoring, considered alongside the cost of sensor network installation and maintenance, and data transmission, storage and processing, establish an economic challenge. Mobile sensing paradigms are perceived to offer a potential advantage here in terms of reduced cost [13] and reduced resource and time requirements [14], allowing sensors to roam across multiple bridge structures in a transport network [15]. This paradigm is known as indirect structural health monitoring (iSHM) in accordance with the abstraction of the sensors from the subject structure. The indirect approach is predicated on the coupled dynamic interaction of a vehicle and a bridge during a traversal. Yang et al. [14] first suggested the recovery of bridge modal frequencies from the vehicle response. Since then, selected recent examples can be used to highlight the current state of the field in pursuit of bridge modal parameter extraction. In this regard, research efforts have included estimation of parameters such as bridge mode shapes [16, 17], operational deflected shape ratios [18] and modal damping ratios [19]. Non-modal damage-sensitive features have also been explored, including works investigating the application of machine learning (for example [20, 21]). Broad enthusiasm from researchers is highlighted by the extent of works presented in recent review papers (for example [22, 23]).

The economic benefits of iSHM are expected to be compounded if the sensing and transmission networks can be effectively outsourced to citizens or private companies. This is known as *crowdsensing* [24] or *crowdsourcing* [25]; the two terms are synonymous. A significant challenge for iSHM is the effect of road surface roughness, which causes additional vibrations that can mask the visibility of bridge-related signal features and ultimately hamper the identification of bridge damage. Some degree of mitigation is offered by approaches which combine time-shifted acceleration responses from multiple axles, for example on towed trailers [26, 16]. In the context of crowdsourcing, similar approaches have been proposed. Some studies identified the benefit of using ensemble methods to address confounders such as vehicle properties (for example [24, 27]). Matarazzo et al. [25] also report an investigation in which acceleration responses from multiple vehicles and traversals were used to counter the effects of vehicle–road–bridge interaction, leading to enhanced visibility of bridge frequencies in the ensemble data. The approach showed promise in field tests on a long- and a short-span bridge.

The use of human-propelled vehicles such as bicycles as sensor carriers is inherently amenable to crowd-sourced data gathering, since many notionally identical vehicles will naturally traverse multiple bridge structures during normal operation, and suitable sensors can be easily mounted (for example, in bicycle hire schemes increasingly seen in urban environments worldwide). It is intuitive that bridges carrying non-motorised traffic can suffer from the same damage-related and EOV issues as highway bridges. To date, there has been only very limited study of the use of alternative vehicle types as sensor carriers. Quqa et al. [28] report a study in which bicycle-mounted smartphones were used to estimate bridge fundamental frequency and operational mode shape, and experiments by Yang et al. [29] featured hand-pulled carts acting as sensor carriers. Otherwise, research in this field to date generally relates to motorised vehicles (cars, buses, trucks) on highway road bridges, or trains on rail bridges. (A recent notable exception is the investigation of electric scooters for this purpose, presented by Li et al. [30, 31], which can also be situated within the framework of crowdsourcing). It is recognised that new paradigms will be required to enable the realisation of indirect bridge monitoring in the future—see for example the paper by Gkoumas et al. [32], which discusses the future application of fleets of autonomous vehicles for this purpose. Collectively, the existing literature suggests that sensors mounted on bicycles could potentially be employed for in-service indirect estimation of bridge modal properties, but that EOV-related challenges are likely to arise. Very few studies have pursued this agenda to date. In response, the current work attempts to extract estimates of the modal frequencies of a bridge in field-scale tests, while exploring the potential for confounding due to variations in environmental conditions and the operating regimes of the bicycle.

## Methodology

This paper presents the results of field investigations combining direct and indirect monitoring of a bicycle/pedestrian traffic bridge subject to pedestrian heel drop impacts, ambient excitation and traversals by bicycle. Both the bridge deck and the bicycle frame were instrumented with wireless accelerometers, simultaneously transmitting data to a common data logger during the traversals. The tests included multiple traversals, allowing the confounding effects of road surface profile roughness to be countered by ensemble averaging. The testing regime included traversals with and without pedalling at two cadences in order that the effects of harmonic load variation due to pedalling dynamics could be explored.

### Assumed theoretical model

Yang et al. [14] abstracted a vehicle (with parameters representative of a car) as a single degree-of-freedom sprung mass model when considering in-plane motion only. They considered the coupled interaction of this vehicle model with a simply supported beam and showed, in closed analytical form, that bridge modal frequencies would be visible in the vehicle response (and vice versa). It has been shown theoretically [33] and practically at laboratory scale [34] that when a vehicle traverses a bridge, vehicle–bridge interaction (VBI) leads to nonstationary frequencies for the combined vehicle–bridge system when their respective natural modal frequencies are proximal. A rigid-frame bicycle (non-suspended) with pneumatic-tyred wheels would appear to be even more amenable to abstraction as a single-degree-of-freedom system than a car. To the authors’ knowledge the phenomenon of frequency nonstationarity has not previously been suggested or explored for human–bicycle–structure interaction.

**Fig. 1:**
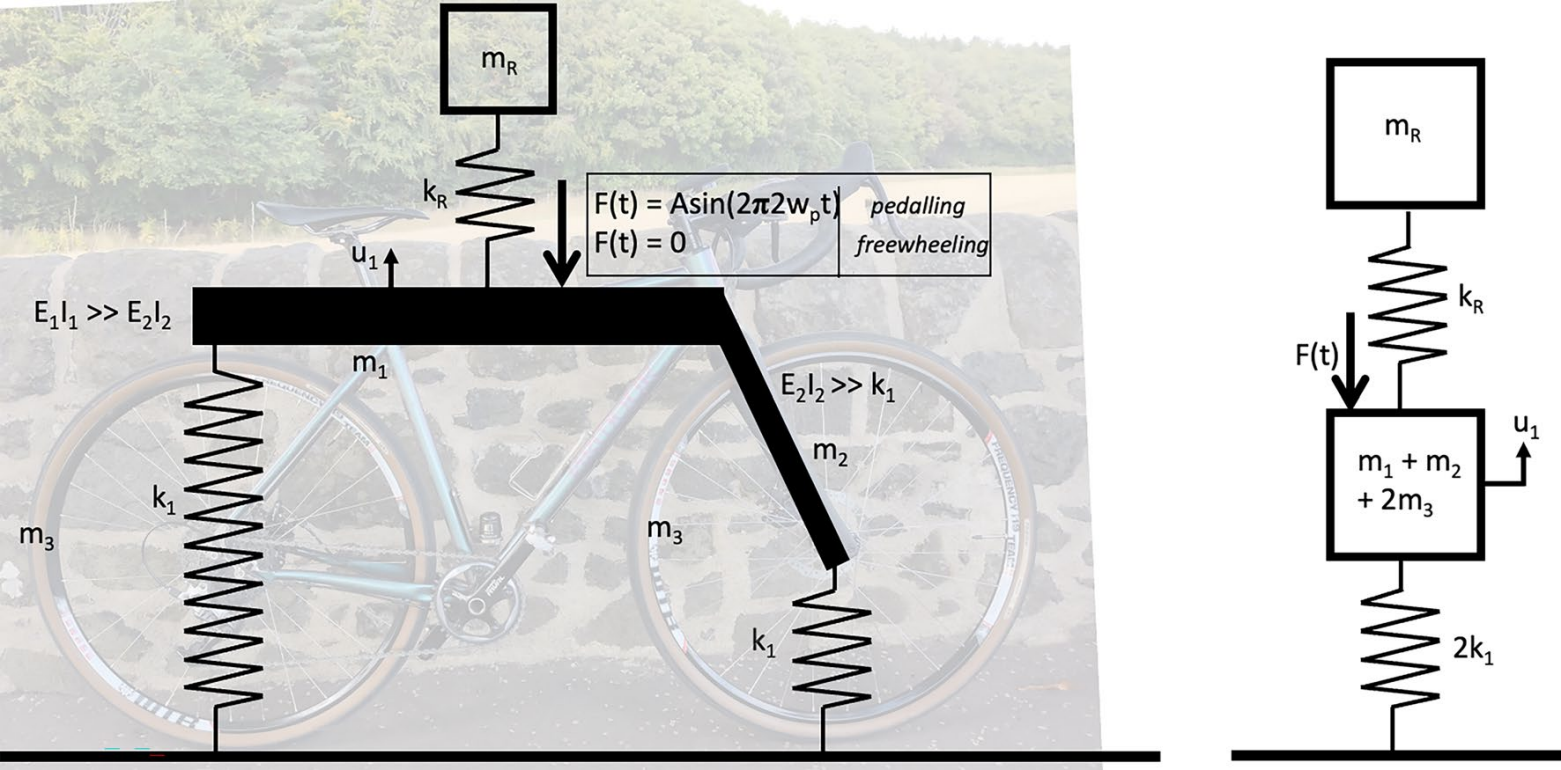
Deriving the assumed reduced degree-of-freedom model of rider and bicycle system. Left: assumed system properties. Right: reduced degree-of-freedom abstraction based on assumed properties

The authors propose that pedalling can be modelled as a harmonic input force acting on the lumped bicycle mass. The use of harmonic excitation in moving vehicles for the purpose of bridge modal parameter estimation was explored by Zhang et al. [35]. Through numerical simulation and laboratory-scale experiment they sought to extract estimates of mode shape squares, hypothesising that these would be sensitive to damage. They observed more accurate results when the exciting vehicle’s harmonic load frequency was close to, but not equal to, the frequency of the structural mode being studied, thus avoiding resonance. In the current work, the bicycle is abstracted as a single-degree-of-freedom (SDoF) sprung mass system with a harmonic oscillator providing a time-dependent driving force during pedalling. The rider is abstracted as a further SDoF system, with the rider–bicycle assembly, thus comprising a two-degree-of-freedom (2DoF) model. The accelerometer is mounted on the bicycle frame approximately centrally between the axles and the vertical motion of the combined rider–bicycle assembly can be approximated as shown in Fig. 1. In this figure, $u_1$ is the vertical displacement at the sensor location; $m_3$ is the wheel mass; $m_2$ the fork mass; and $m_1$ the mass of the rest of the bicycle. $m_R$ represents the mass of the rider. $k_1$ is the effective spring stiffness of each tyre, $E_1I_1$ and $E_2I_2$ are the effective bending stiffnesses of the bicycle frame and fork, respectively, and $k_R$ represents the effective spring formed by the rider’s body.

The bicycle frame is a triangulated structure whose vertical stiffness will be very high. The bicycle’s fork is a cantilever structure, but it is nevertheless assumed to be very stiff in relation to the tyres. Thus, the effective spring stiffness supporting the lumped bicycle mass (Fig. 1) is assumed to be dominated by the tyres and thus will be related to tyre inflation pressure, tyre volume and—at lower pressures—tyre casing sidewall stiffness. Early studies of vehicle–bridge interaction commonly disregard the effect of vehicle suspension damping as part of an overall aim to simplify the abstracted physical model (for example, the SDoF sprung mass model used by Yang et al. [14]). This was presumably adopted in order to simplify the coupled dynamic model of vehicle and bridge to facilitate a closed-form analytical solution, or based on the assumption that damping is of negligible importance in transient dynamic interactions as noted by Yang et al. in their 2019 book [36]. Although later (typically numerical) studies adopt models including suspension damping, tyre hysteresis is still commonly omitted (see for example the two degree-of-freedom model used by Corbally and Malekjafarian [37]).

Representing the bicycle as a single-degree-of-freedom system (the lower mass on spring indicated in Fig. 1) implies that only vertical motion of the bicycle will be considered. Other motion, both in-plane (pitch) and out of plane (roll and yaw) will not be captured by a single accelerometer recording the vertical response. The adopted simplified representation of the bicycle is based on the assumption that vertical acceleration related to these motions will be small and can therefore be neglected. Considering in-plane motion, the adopted accelerometer mounting position (approximately central between the axles) means that the amplitude of any pitch motion should be low at the sensor location. Out of plane, roll angles are necessarily small as the bicycle remains approximately upright during forward travel in a straight line. Yaw should be negligible assuming that no slipping occurs between the tyres and roadway.

Figure 1 also indicates that the rider is represented as a lumped mass supported on a spring. Limebeer and Sharp [38] report that representing the rider’s mass as a single point with rigid connection to the bicycle frame was common in earlier studies of bicycle dynamics and control, certainly those following the approach outlined by Whipple [39]. The lumped sprung mass approximation is common in studies of human–bridge interaction as summarised by Caprani and Ahmadi [40] who report that a range of mass and spring stiffness values for such a single-degree-of-freedom (SDoF) abstraction has been proposed in the literature. Zhang et al. [41] (from Caprani and Ahmedi [40]) reported frequencies for such sprung mass models in the range 1.78–1.92 Hz. Recently, some authors have investigated the transmissibility of vibration to the rider as a function of rider posture [42]. However, in their study pedalling cadence was zero: the cranks were stationary and horizontal. Viellehner and Potthast [43] explored the effect of vibration on muscle activation in cyclists and found a relationship varying with crank cycle phase, differing between the upper and lower parts of the cyclists’ bodies. To the authors’ knowledge, cyclist posture and pedalling cadence have not been investigated specifically in relation to effective frequency of an SDoF model. In the present work, it is assumed that pedalling cadence will influence the effective modal frequency of the abstracted SDoF representation of the cyclist. Two cadences are explored and a harmonic pedalling force is assumed to act on the lumped mass representing the bicycle, with $F(t) = Asin(2\pi 2w_pt)$) where $w_p$ is the circular pedalling frequency, or cadence, and the multiplier 2 applied to $w_p$ accounting for the fact that two downward forces will be realised (one from each pedal) for one full rotation of the cranks in the bicycle drivetrain. The bicycle and rider combined system is assumed to traverse the bridge at a fixed velocity *v*, with the harmonic exciting force defined as $F(t) = 0$ for the freewheeling case.

**Fig. 2:**
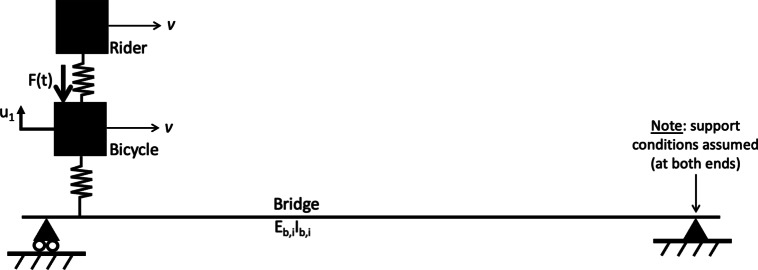
Assumed rider–bicycle–bridge interaction model

**Fig. 3:**
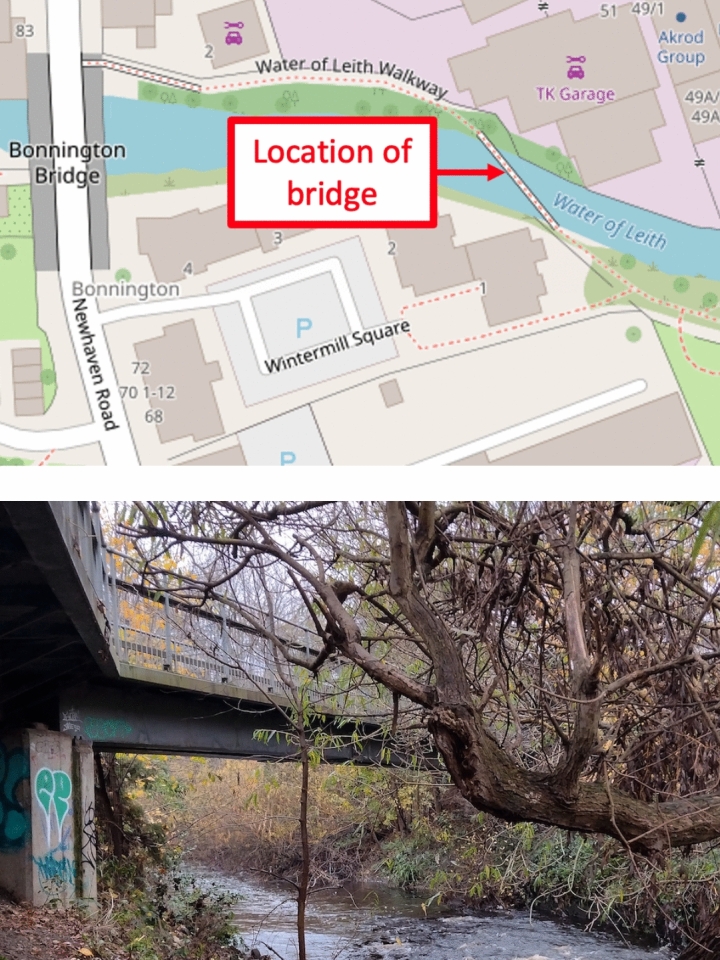
Upper part: bridge location plan (orientation north up). Map ©OpenStreetMap contributors, available under the Open Database Licence [44]. Lower part: site photograph looking approximately west

The bridge structure with length $L = 30$m is assumed to be a simply supported beam with bending stiffness $E_bI_b$ as indicated in Fig. 2, which presents the assumed rider–bicycle–bridge interaction model. The subject bridge forms part of a public shared pedestrian/bicycle path in Edinburgh, Scotland and is located as shown in Fig. 3 alongside an illustrative photograph.

### Experimental regimes

**Fig. 4:**
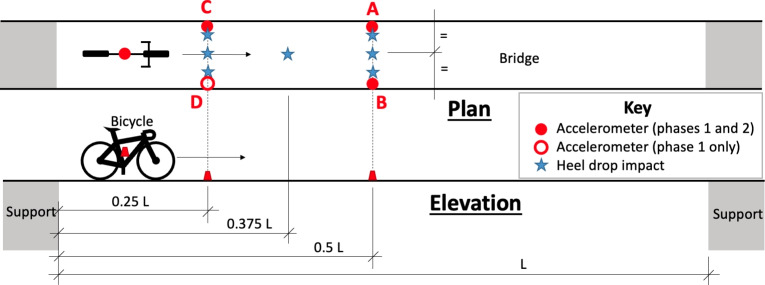
Schematic layout indicating sensor placement on bike and bridge during traversals, and location of accelerometers and pedestrian heel drop impacts during *phase 1* of testing

The fieldwork was split into two distinct phases which were conducted on different days. It should be noted that phases 1 and 2 are named for the logical order of the work undertaken, i.e. baseline system identification in the first phase and field trials of bicycle traversals in the second phase. However, in calendar terms, phase 2 was actually undertaken first. In phase 1 (undertaken on 15th December 2022), the bridge was excited by pedestrian loading. The load was applied by so-called *heel drops* (a person raising onto the balls of their feet, then dropping their heels simultaneously to the ground), thus approximating an impulse load from bodyweight at varying locations. Between heel drops, a minimum pause of around 10 s was allowed such that the induced amplitude of bridge vibration would be damped out. The sensor and impact locations are shown in Fig. 4 and summarised in Table 1.

In phase 2 (undertaken on 2nd December 2022) the rider–bicycle system traversed the bridge in both pedalling and freewheeling states. Two distinct pedalling cadences were tested. The two regimes achieved distinct cadences by the use of different gear ratios. The bicycle was equipped with a single front (38-tooth) chainring and an 11-speed rear cassette. The *slow-* and *fast-cadence* regimes used sprockets 5 (19 teeth) and 9 (32 teeth), respectively, abbreviated as *spr. 5* and *spr. 9* in the remainder of the work. The ratio of cadence between the two regimes was therefore around 1.68. The exact pedalling cadence was not recorded. The traversals are summarised in Table 2. The traversal speed was estimated by picking peaks in the bicycle-mounted sensor’s acceleration response, taken to correspond to the bicycle passing over the deck joints as it entered and exited the bridge span. This resulted in minimum and maximum traversal speeds of 3 m/s and 5 m/s, respectively. The mean traversal speed was 4.25 m/s and the median was 4.29 m/s.

In both phases 1 and 2, a period of bridge response to ambient conditions was also recorded. The total number of data samples, the sensor locations and the sample lengths used across all of the regimes are summarised in Table 3.

**Table 1:** Heel drop impact locations and quantities recorded in phase 1

Impact location	No. of heel drops
Midspan central	27
Midspan eccentric (B)	12
Midspan eccentric (A)	18
1/4 span central	14
1/4 span eccentric (D)	18
1/4 span eccentric (C)	12
3/8 span central	14

**Table 2:** Bicycle traversal regimes and quantities recorded in phase 2

Regime	No. of traversals	Bicycle gear ratio (chainring x sprocket)
Freewheeling	13	–
Pedalling: slow cadence (spr. 5)	10	38 × 19
Pedalling: fast cadence (spr. 9)	3	38 × 32

**Table 3:** Sample quantity, sensor locations and sample lengths used

Type of input	Sensor locations	Total number of samples per sensor	Sample length
Heel drops (phase 1)	Bridge	115	6 s
Ambient (phases 1 and 2)	Bridge	2	40 s
Bicycle traversals (phase 2)	Bridge and bicycle	26	Mean 7.21 s

### Sensor instrumentation

The data was gathered using LORD Microsystems G-Link-200 8G wireless triaxial micro-electromechanical system (MEMS) accelerometers. The sensors were magnetically fixed to the bicycle and bridge deck in the locations indicated in Fig. 4. The magnetic sensor bases were sufficiently strong to adhere to the ferrous structure of the bridge through the non-structural finish layer on the structure.

**Fig. 5:**
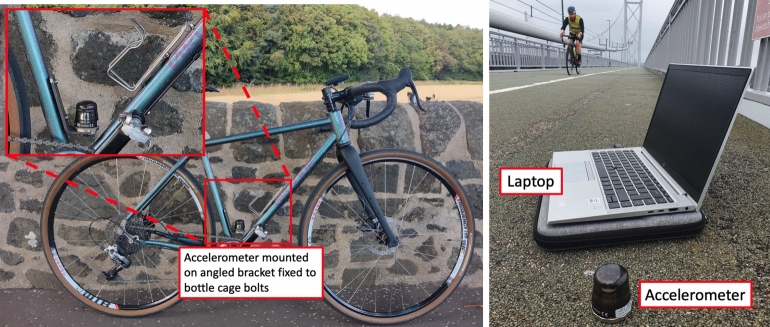
Left: the bicycle used for data gathering, showing wireless accelerometer installed. Right: example of the first author riding the bike, with wireless accelerometer magnetically fixed to (a different) bridge deck and data logging laptop computer visible in foreground (photo by Mr Thomas McCormick)

The sensor installed on the bicycle is shown in Fig. 5 along with an illustration of the bicycle in use with the data logging laptop visible. Data were logged at a sampling frequency of 512 Hz for phase 1 and phase 2. No frequency or other filtering was employed, other than the sensors’ built-in analogue antialiasing filters (at 1.5 kHz) and digital downsampling to achieve the specified sampling rate. Data was transmitted wirelessly in so-called *calibrated* format, meaning that factory pre-set calibration factors were applied automatically.

### Data processing (impulse response)

**Fig. 6:**
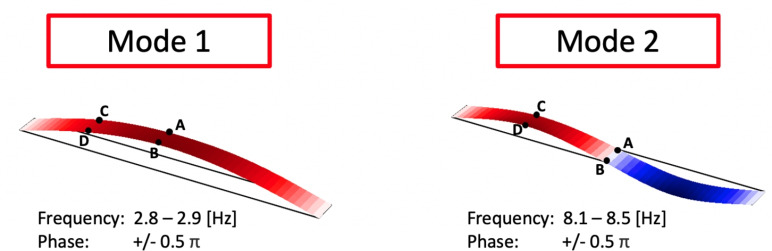
Three-dimensional mode 1 (left) and 2 (right) shapes estimated using the matrix pencil method applied to bridge-mounted sensors (locations shown indicatively), excited by pedestrian loading (heel drops)

The data collected during phase 1 were used to estimate bridge modal parameters from each sensor’s response to the applied heel drop impacts. The identification process reported in this section was based only the responses from accelerometers attached directly to the bridge. The matrix pencil method (MPM) was adopted and used to generate estimated modal frequencies, damping ratios, phase angles and amplitudes for the first two global bending modes of the bridge. The estimated modal amplitudes are related to acceleration amplitude and represent modal participation for each sensor location, thus allowing esimation of unscaled mode shapes. The MPM was proposed by Hua and Sarkar [45] and is a time-domain curve-fitting paradigm which uses each sensor’s response to an impulse load to estimate modal parameters (frequency, amplitude and phase angle). Frequency bands were identified manually by inspection of the estimated frequency–damping pairs. For these bands, the modal amplitudes were used to estimate mode shapes. Estimated mode shapes and frequencies are shown in Fig. 6. Mode shapes were generated based on modal estimates within the frequency limits noted in Fig. 6. Phase coherence was enforced by including only estimates within the phase angle ranges noted in Fig. 6. Modal estimates with damping in excess of 5% were assumed to be spurious and were therefore discarded. For mode 1, symmetry about the bridge midspan was assumed. For mode 2, antisymmetry about the midspan was assumed.

### Data processing (ambient response)

Periods of ambient response data from phase 1 and phase 2 were used to estimate bridge modal parameters, using only the data from accelerometers fixed directly to the bridge. Initially the phase 2 data were used and the covariance driven stochastic subspace identification (SSI-COV) method was adopted. The SSI-COV method used here was originally proposed by Magalhães et al. [46] who explain that it involves the use of singular value decomposition of the covariance matrices of the time series of structural response, followed by using least-squares optimisation to fit to a state-space representation of the structural behaviour. This implementation of SSI-COV was used by Cheynet et al. to estimate bridge modal parameters based on response to ambient excitation [47] and their MATLAB code [48] has been adopted in the current study for data processing and generation of plots (Figs. 9 and 10) showing estimated modal parameter outcomes.

Ambient response data from bridge-mounted sensors in phases 1 and 2 were also subsequently used to generate estimates of power spectra using Welch’s method [49] which comprises frequency-domain averaging of overlapping windowed segments of the signal. These are compared in Fig. 12 and discussed in Sect. 3.2.

### Data processing (response to bicycle traversals)

The traversal response data from phase 2 (from bridge- and bicycle-mounted sensors) was split into subsamples in the time domain corresponding to each bike traversal. An ensemble average of the traversals for each experimental configuration was then used to generate an estimate of the power spectral density using Welch’s method, for which the open source Python library SciPy [50, 51] was used. Various other open source Python libraries were also utilised.

## Baseline bridge modal identification

### Pedestrian heel drop excitation: three-dimensional mode shapes

**Fig. 7:**
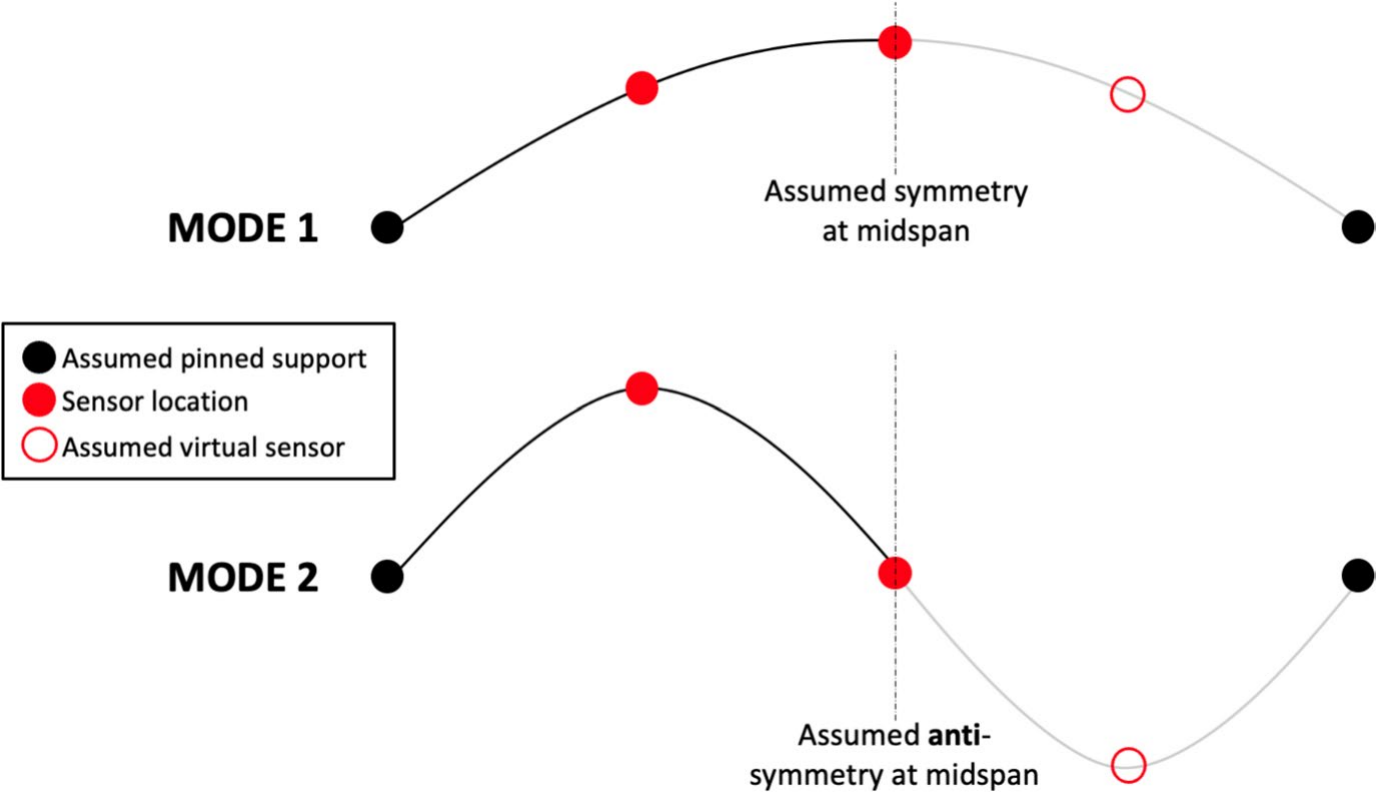
Illustration of sensor locations, assumed virtual sensors and assumed support conditions used in estimation bridge mode shapes

**Fig. 8:**
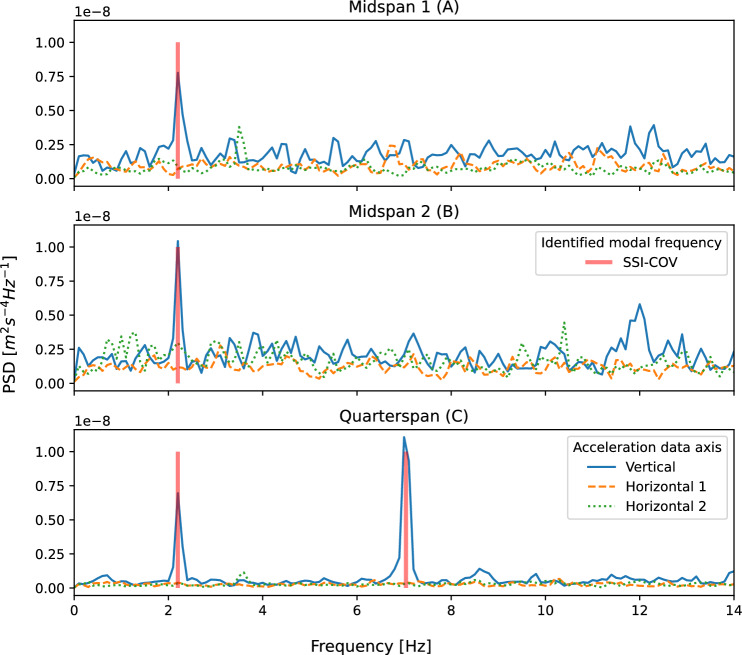
Ambient response in three dimensions for *phase 2*

The first two three-dimensional bridge modes estimated and extrapolated from pedestrian heel drop loading in phase 1 are shown in Fig. 6, based on applying the MPM to the bridge acceleration response recorded at sensor locations A, B, C and D (Fig. 4) and modelling the bridge in three dimensions. Zero displacement was assumed at the supports. A virtual sensor at the three quarter-span point was assumed based on the motion recorded at the quarter-span point, either in-phase or $\pi$ radians out of phase according to the mode shape studied. This can be understood as an assumption of symmetry or anti-symmetry at midspan for the first and second bending modes, respectively. The real and virtual sensor locations, and assumed support conditions for these two modes are illustrated in Fig. 7. Cubic interpolation was used between the sensor locations. It was assumed that no lateral or torsional modes were active during this experimental regime, despite the loading including longitudinally asymmetric impulses. This assumption was confirmed by inspection of the power spectra of ambient response in three dimensions from phase 2, as shown in Fig. 8; the first two frequencies appeared as distinct peaks in the vertical direction, while in the horizontal directions no clear peaks were apparent. The phase coherence requirements noted in Fig. 6 provide additional reassurance that the frequency deemed to represent the first global bending mode did not contain a significant torsional response, since torsion would be expected to manifest with a a greater phase lag between midspan sensor locations A and B. Therefore, only vertical acceleration responses were used. The assumption of the bridge acting as a simply supported beam was considered to be reasonable, although the potential for varying behaviour due to the true nature of the support conditions is yet to be explored in detail as highlighted in Fig. 2.

### Comparing pedestrian heel drop and ambient excitation: two-dimensional mode shapes

**Fig. 9:**
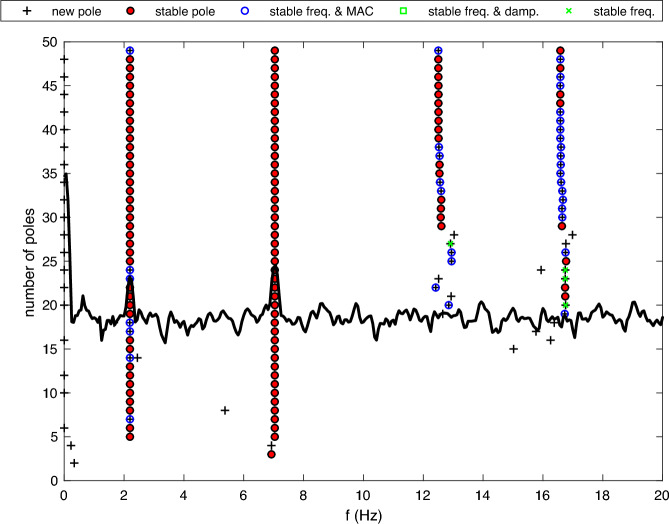
Stabilisation diagram for modal estimates using SSI-COV (ambient excitation)

Figure 9 presents the stabilisation diagram for the first two bridge modes estimated from the quarter-span sensor (location C in Fig. 4) using the SSI-COV method (phase 2). The two identified modal frequencies are evident as prominent peaks in the frequency domain representation of the sensor response to ambient excitation, and appear consistently stable across a range of potential model orders.

**Fig. 10:**
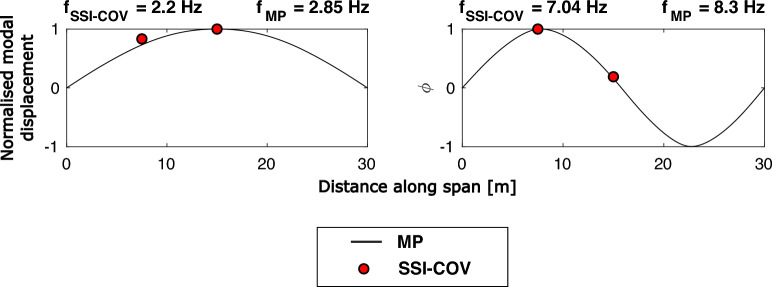
Comparison of mode shapes estimated using SSI-COV (ambient excitation) and matrix pencil (MP) method (heel drop excitation)

A comparison of the estimated mode shapes from the MPM and SSI-COV methods is presented in Fig. 10. Since no significant torsional response was observed when considering the response to pedestrian heel drop inputs, the comparison presented in this section models the bridge as a two-dimensional line-type structure. The MPM mode shapes are indicated as lines, based on the response at sensor locations A and C (Fig. 4) and the interpolation strategy discussed in Sect. 3.1. For ease of visual comparison, the SSI-COV modal amplitudes at the same sensor locations are indicated as single points in Fig. 10. Good agreement is evident, confirming that the bridge structure appears to act as a beam-like structure for its first two modes under impulse (heel drop) and ambient excitation regimes. However, the modal frequencies estimated by the SSI-COV method are consistently lower for both modes. Table 4 compares these frequencies alongside the range of ambient temperature for each day of fieldwork, from weather records provided by the website Visual Crossing [52]. Other than temperature, weather conditions were qualitatively deemed to be similar between the two days of fieldwork.

**Table 4:** Estimated bridge modal frequencies and daily ambient temperature ranges for the two phases of fieldwork, grouped according to excitation input type

Excitation source	Bridge modal identification method	Ambient daily temperature range [°C]	$f_{B1}$ [Hz]	$f_{B2}$ [Hz]
Heel drops (phase 1)	MPM	$-$6.1 to +1.0	2.85	8.3
Ambient (phase 2)	SSI-COV	+6.0 to +8.7	2.2	7.04

**Fig. 11:**
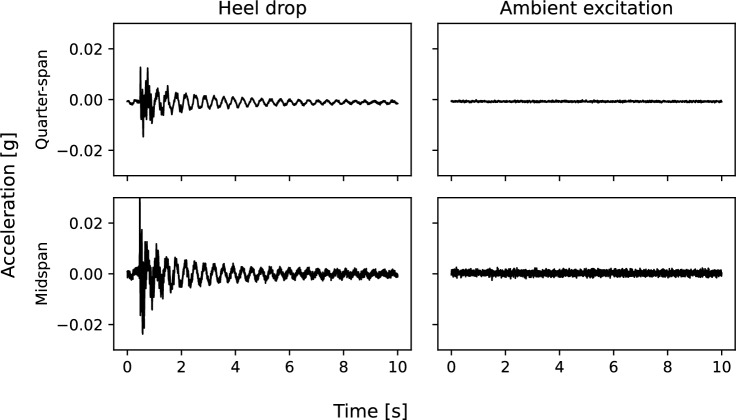
Illustrative comparison of acceleration response amplitudes

During phase 1, the ambient temperature was reportedly lower [52] than in phase 2. It is plausible that temperature reduction could have caused a stiffening effect (for example to the bridge bearings), leading to increased frequencies identified for the corresponding modes in response to heel drop excitation. On the other hand, Fig. 11 presents an illustrative demonstration that acceleration response amplitudes were higher for the heel drop excitation regime. It is also plausible that the higher frequencies associated with this phase of experimentation could represent amplitude-related frequency variation for the bridge structure. Other than temperature and response amplitude the other notable difference between the two experimental system identification regimes is the presence of a person on the bridge deck. It is therefore plausible that the presence of a person on the bridge deck during the heel drop regime could have influenced the observed bridge frequencies, at least for the first bridge mode. The addition of mass is well known to cause a frequency decrease, but in this case was correlated with an observed increase in bridge frequency. In relation to the bridge system identification exercise it was therefore assumed that in this case the added mass was negligible, with the observed frequency change associated with either the ambient temperature or the amplitude of acceleration response of the bridge deck, rather than by the presence of a person on the deck.

**Fig. 12:**
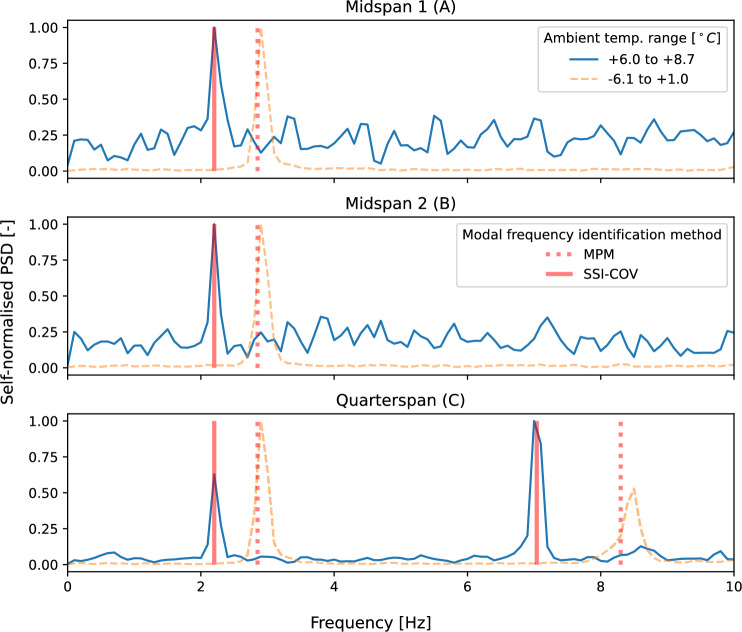
Self-normalised power spectra, estimated using Welch’s method, for ambient bridge response. Three sensor locations and two ambient temperatures are compared

Although the potential causes of frequency change noted above are all plausible, an alternative explanation would be that the frequency change was an artefact of the differences in estimation methods employed (MPM and SSI-COV). To discount this, ambient responses from both phases of fieldwork were compared. In each case, 40 s of recorded data were used. The outcomes, shown in Fig. 12 compare the self-normalised power spectra estimated for sensor locations at midspan (A and B) and quarter-span (C) on the bridge. The frequency peaks in the estimated spectra appear to correspond to the estimated frequencies for the first two modes, according to the ambient temperature (Table 4). This demonstrates that ambient temperature—rather than the method of parameter estimation—appears to be the cause of the frequency shift. The bridge frequencies estimated by SSI-COV are therefore deemed to represent the bridge parameters relevant for the day of bicycle traversals (phase 2).

## Bicycle traversals

**Fig. 13:**
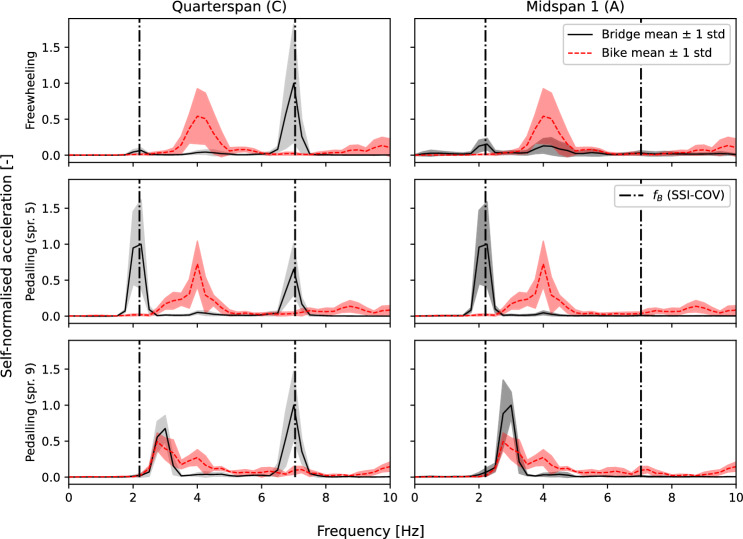
Power spectral densities for bike and bridge sensors. Ensemble averages, self-normalised

Figure 13 shows the power spectral density estimated using Welch’s method for bridge and vehicle sensor responses from traversals (phase 2). The traversal data are presented as a self-normalised ensemble average. To achieve this, the signal power for each traversal was estimated and stored as a row vector. An element-wise average was then calculated for the ensemble of all vectors corresponding to each traversal regime (freewheeling, pedalling at slow cadence (*spr. 5*) and pedalling at fast cadence (*spr. 9*) in turn. The average signal power vector for each regime was then normalised to its own maximum value. The ensemble average self-normalised data were then plotted with their mean plus or minus one standard deviation indicated across the displayed frequency range. Vertical lines indicate the bridge modal frequencies estimated (Sect. 3) by SSI-COV in a dash–dot line type. The data are grouped into three distinct regimes: freewheeling (upper plot); pedalling at slow cadence (middle plot); and pedalling at fast cadence (lower plot). The duration was approximately equal for all traversals. A comparison is presented between the bicycle-mounted sensor response and the bridge quarter-span (left subplots) and midspan (right subplots) sensor locations.

Inspection of the left subplots in Fig. 13 reveals a clear peak in the bridge quarter-span sensor response at the second mode frequency identified by SSI-COV. This peak is clearly visible for all bicycle traversal regimes, suggesting that the presence of the bicycle did not affect the bridge second bending mode. Conversely this modal frequency does not appear to be visible from the bicycle-mounted sensor.

Both bridge sensor locations appear to show a peak in the region of the identified bridge first mode frequency (SSI-COV) under freewheeling and slow cadence pedalling regimes. In these regimes, the bicycle sensor response shows a peak around 4 Hz. During bicycle traversals with a higher pedalling cadence, the bridge and bicycle sensor responses appear to show convergence of their respective frequency peaks to just below 3 Hz. The greatest variation (largest standard deviation) within the frequency range shown in Fig. 13 is associated with the peak around 4 Hz in the bicycle-mounted sensor response in the freewheeling regime.

**Fig. 14:**
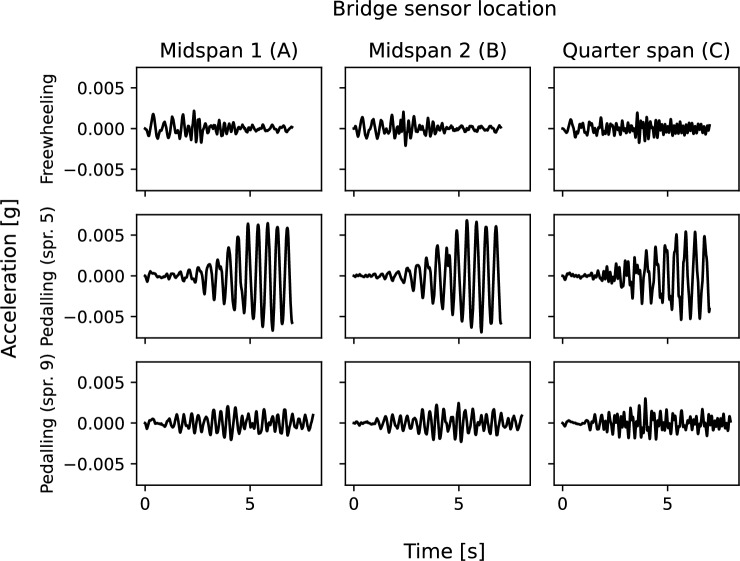
Bridge acceleration responses for sample traversals: time domain

The ensemble average spectra presented in Fig. 13 were self-normalised but appeared to indicate differing signal power associated with frequency peaks for each of the freewheeling and pedalling cadence regimes. To explore this further, Fig. 14 compares the time-domain bridge sensor responses for a sample traversal in each of the three explored regimes. Prior to plotting, the signals were subject to a frequency band-pass filter approximately centred on the bridge first mode frequency. An increasing response amplitude, with its highest magnitude close to the end of the traversal is associated with the slower pedalling cadence (middle row of subplots) implying that resonance was induced by approximate matching of harmonic pedalling force with this bridge frequency. The freewheeling and faster pedalling cadence regimes do not show such increasing response amplitude, further implying that the phenomenon is related to resonance of the bridge with the harmonic pedal force rather than any other bridge or rider–bicycle system parameters.

**Fig. 15:**
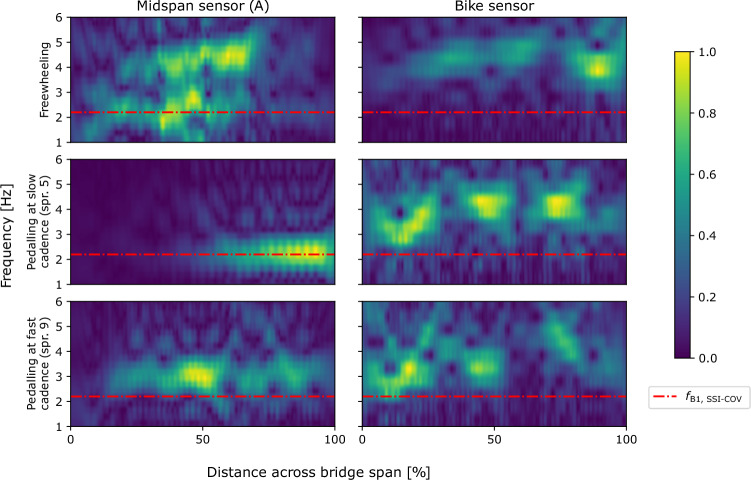
Bike and bridge acceleration responses for sample traversals: time–frequency domain

Proximity of modal frequencies between the bicycle–rider system and the bridge could lead to a shift in the measured frequency during traversals. The assumed 2DoF rider–bicycle model shown in Fig. 1 would have two associated modal frequencies. If the lower spring is far more stiff than the other ($2k_1>> k_R$), then one of the modal frequencies would tend towards $f_{BR} \approx \sqrt{k_R / m_R}$ as noted by Jazar for a 2DoF quarter car model [53]. Recalling the frequencies presented for SDoF models of humans (1.78–1.92 Hz, [41] from Caprani and Ahmedi [40]) and the bridge first mode frequencies estimated by MPM (2.85 Hz) and SSI-COV (2.2 Hz) it seems plausible that the bicycle–rider and bridge systems could have proximal first mode frequencies (i.e. one of the bike-rider system frequencies $f_{BR}$ is approximately equal to the first bridge natural frequency $f_{B1}$. In situations of approximate frequency matching, combined system frequencies are expected to be nonstationary during traversals. To explore this, Fig. 15 presents time-frequency contour plots for an illustrative sample set of traversals for all three regimes. The plots were generated by using the short time Fourier transform implementation in SciPy [54] and the contours are self-normalised (to the maximum signal power at each timestep) to allow comparison of the trends of frequency variation with time. In the freewheeling regime, two frequencies are visible and their approximate pattern variation matches that expected based on prior literature [33] if $f_{BR} \approx f_{B1}$. The same trend is visible in the data from the bicycle-mounted sensor for freewheeling and slow pedalling cadence regimes. The magnitude of the greatest frequency shift (expected when the bicycle–rider system is at the bridge midspan) would be determined by the relative mass ratio between the rider–bicycle and bridge systems; since this mass ratio is not known, the expected trend was not plotted. For the faster pedalling cadence regime, the trend is no longer clearly apparent. A potential explanation is that when the rider was more relaxed (freewheeling and slow pedalling cadence regimes) the proposed frequency matching was present, but the rider’s arms and upper body were stiffened in order to stabilise their posture while pedalling at a faster cadence, resulting in greater spacing between the frequencies ($f_{BR}> f_{B1}$) thus reducing the nonstationary behaviour effect.

## Discussion

The data gathered to date suggest that bicycles may have a potential application as sensor carriers for indirect structural health monitoring of bridges, but that EOV remains a challenge. In the freewheeling regime, bridge first mode frequencies estimated from the bicycle-mounted sensor were shifted from those shown by direct-mounted sensors. In the slow pedalling cadence regime, this shift was still apparent and was accompanied by an increase in the amplitude of the bridge first mode response due to resonance. The fast pedalling cadence regime resulted in convergence of the first mode frequency estimated from bicycle- and bridge-mounted sensors, but the estimated frequency was still shifted (albeit less so for this regime) from the prior identified baseline.

Subject to bridge natural frequencies and pedalling cadence falling within favourable ranges (i.e. $2w_p \approx f_n$ for bridge mode $n$), it appears that pedalling may enhance bridge mode 1 response. It is encouraging to note that the bridge mode 1 frequency is still visible from the bridge-mounted sensors when the bicycle is freewheeling, implying that the visibility of this frequency peak for directly mounted sensors is genuine rather than a spurious artefact caused by rider–bicycle interaction, pedalling cadence harmonics or other factors. However, if the pedalling cadence is close to the bridge modal frequency, resonance would be expected and was shown to occur here. If the effective natural frequency of the rider–bicycle system is close to that of the bridge first mode ($f_{BR} \approx f_{B1}$), this appears to lead to nonstationary frequency variation during traversals.

Pedalling at a higher cadence was observed to be associated with a reduction in this nonstationary frequency behaviour. In relation to this, and to the frequency shifts noted above, the dynamics of rider–bicycle interaction were considered. Prior research has suggested that rider posture and pedalling dynamics affect vibration transmission and muscle activation to and of the rider respectively. The observations in the present work suggest that posture and cadence influence the effective frequency of the rider–bicycle system and thus can potentially lead to nonstationary frequency variation of the combined rider–bicycle–bridge system during traversals. Without knowledge of this, the frequency shift could be misinterpreted as representative of bridge damage; in other words, rider–bicycle dynamics are a source of EOV. Greater understanding of these phenomena would increase the utility of crowdsourced data gathered from fleets of bicycles in the future. Cadence measurement is already standard for sports bicycles, albeit at low resolution, suggesting the potential for mitigating this aspect of EOV to some degree if an underlying relationship between cadence and effective frequency can be demonstrated. Further research is recommended.

Two methods of baseline bridge modal identification were compared; they yielded differing frequency estimates for the first two bridge modes. It was noted that these frequency differences could plausibly relate to either temperature effects or response amplitude effects. Alternatively the difference could have been an artefact of the differences in estimation methods used. To explore this a further comparison was made using a third estimation method applied to ambient bridge responses (i.e. with nominally equal amplitudes) from each of the two phases of fieldwork. The outcome suggested that the observed frequency changes were indeed temperature induced.

The following novel contributions are suggested:

- Using field investigation it was shown that low-cost sensor-carrying human powered vehicles can excite and in some situations potentially record bridge frequencies—supporting the only known paper [28] pursuing this specific subject to date.
- Known prior work [28] measured the bridge under ambient conditions and compared to bicycle-mounted sensor response during traversals at a later time. This paper adopted simultaneous sensing of vehicle and bridge, reducing potential ambiguity.
- The present work is the first known demonstration of nonstationary frequency behaviour due to assumed frequency matching between the rider–bicycle and bridge, a phenomenon that has previously only been discussed and explored for large vehicles.
- The investigations presented highlight the potential confounding effects of rider–bicycle and human–structure interaction on iSHM using human-powered vehicles. The outcomes suggest that rider posture and pedalling cadence are important factors relevant to the accuracy of extraction of bridge frequencies from the bicycle-mounted sensor response.
- The work pursues indirect bridge modal parameter estimation without the need for motorised vehicles, supporting a general societal need to decarbonise as well as extend the life of existing infrastructure and contributes to addressing the general limited quantity [22] of field-scale testing of indirect monitoring methods.

Some specific limitations of the presented work should be highlighted. The frequency of pedalling cadence in the two regimes was not accurately recorded. Repeating the experiments with an independent way of recording this would allow further insight and improve reliability (for example, proximity of pedalling harmonic to bridge modal frequencies) and could be implemented in future tests. Additionally, the pedalling force was assumed to be harmonic, but may in fact be more complicated. It would also be credible to assume that the force varied as a half-sine, with $F(t) = 0.5A[sin(2\pi 2w_pt) + |sin(2\pi 2w_pt)|]$. Measurement of the applied pedalling force, either in situ (perhaps with pedal-based or crank-based load cells) or in a laboratory setting, would inform future studies by allowing more accurate characterisation of the equivalent force spectra imposed upon the bridge during traversals.

## Conclusions and future work

Detection of the first mode frequency of a bridge from a bicycle-mounted accelerometer has been pursued and convergence of frequencies from bicycle- and bridge-mounted sensors was demonstrated for the fast cadence pedalling regime. Simultaneous sensing on the traversing bicycle and subject bridge structure was employed to reduce possible ambiguity of indirect frequency extraction. The outcomes suggest that the bicycle-mounted sensor paradigm may have potential for iSHM campaigns across a large number of lightweight pedestrian and cycle bridges worldwide, but that confounding due to EOV relating to rider–bicycle interaction dynamics should be considered alongside the effects of environmental factors such as temperature.

Baseline bridge identification was noted to feature temperature-dependent frequencies in accordance with the EOV effects noted in prior literature.

Nonstationarity of rider–bicycle–bridge combined system frequencies was observed during traversals for the first time. Such effects must be taken into account to avoid spurious identification of bridge damage due to apparent frequency shifts using iSHM methods. Observed bridge frequencies and the evolution of bridge acceleration response amplitudes during traversals appeared to show dependence on rider pedalling cadence and posture, the former of which has not previously been widely explored in regard to vibration transmission from bicycle to rider.

Future research is warranted to validate the findings on a second bridge with differing modal parameters. Utilising a variety of gear ratios, it is intended to extend the study of the effects of varying pedalling cadence and rider posture alongside varying the traversal speed (pedalling and freewheeling), allowing the excitation of higher bridge modes to be pursued as well as further confirming the combined rider–bicycle–bridge system behaviour. Future fieldwork should also explore the effect of tyre inflation pressure (effective vertical spring stiffness) on rider–bicycle–bridge interaction in terms of frequency ratios, pedalling force transfer, and potential signal pollution due to road surface roughness. Future work could also include enhanced sensor instrumentation. In addition to the speed and pedalling cadence data noted above, the assumed representative bicycle model could be challenged and validated with the use of additional accelerometers and gyroscopes, with consideration given to instrumentation on the rider as well as the bicycle to further characterise their combined kinematics.

## Data Availability

The data used in this paper can be downloaded from the following link: https://datashare.ed.ac.uk/handle/10283/8908.

## References

[1] Argyroudis S, Hofer L, Zanini M, Mitoulis S et al (2019) Resilience of critical infrastructure for multiple hazards: case study on a highway bridge. In: ICONHIC 2019 2nd international conference on natural hazards and infrastructure, pp 23–26

[2] Worden K, Farrar CR, Manson G, Park G (2007) The fundamental axioms of structural health monitoring. Proc R Soc A Math Phys Eng Sci 463(2082):1639–1664

[3] Rytter A (1993) Vibrational based inspection of civil engineering structures. Dept. of Building Technology and Structural Engineering, Aalborg University, Denmark. Ph.D.-Thesis defended publicly at the University of Aalborg, p 206

[4] Moughty JJ, Casas JR (2017) A state of the art review of modal-based damage detection in bridges: development, challenges, and solutions. Appl Sci 7(5):510

[5] Fan W, Qiao P (2011) Vibration-based damage identification methods: a review and comparative study. Struct Health Monit 10(1):83–111

[6] An Y, Chatzi E, Sim S-H, Laflamme S, Blachowski B, Ou J (2019) Recent progress and future trends on damage identification methods for bridge structures. Struct Control Health Monit 26(10):2416

[7] Lynch JP (2007) An overview of wireless structural health monitoring for civil structures. Philos Trans R Soc A Math Phys Eng Sci 365(1851):345–372 10.1098/rsta.2006.1932 17255043

[8] Farrar CR, Doebling SW, Cornwell PJ, Straser EG (1996) Variability of modal parameters measured on the Alamosa Canyon Bridge. Technical report, Los Alamos National Lab. (LANL), Los Alamos

[9] García Cava D, Avendaño-Valencia LD, Movsessian A, Roberts C, Tcherniak D (2022) On explicit and implicit procedures to mitigate environmental and operational variabilities in data-driven structural health monitoring. In: Structural health monitoring based on data science techniques, pp 309–330

[10] Sohn H (2007) Effects of environmental and operational variability on structural health monitoring. Philos Trans R Soc A Math Phys Eng Sci 365(1851):539–560. 10.1098/rsta.2006.1935 10.1098/rsta.2006.1935 17255051

[11] Cross E, Manson G, Worden K, Pierce S (2012) Features for damage detection with insensitivity to environmental and operational variations. Proc R Soc A Math Phys Eng Sci 468(2148):4098–4122

[12] Peeters B, De Roeck G (2001) One-year monitoring of the z24-bridge: environmental effects versus damage events. Earthq Eng Struct Dyn 30(2):149–171

[13] Malekjafarian A, McGetrick PJ, OBrien EJ (2015) A review of indirect bridge monitoring using passing vehicles. Shock Vib 1:286139. 10.1155/2015/286139

[14] Yang Y-B, Lin C, Yau J (2004) Extracting bridge frequencies from the dynamic response of a passing vehicle. J Sound Vib 272(3–5):471–493. 10.1016/S0022-460X(03)00378-X

[15] Locke W, Redmond L, Schmid M (2022) Experimental evaluation of drive-by health monitoring on a short-span bridge using oma techniques. In: Dynamics of civil structures, volume 2: proceedings of the 39th imac, a conference and exposition on structural dynamics 2021. 10.1007/978-3-030-77143-0_12. Springer, pp 109–127

[16] Yang Y, Cheng Q, Zhu Y, Wang L, Jin R (2020) Feasibility study of tractor-test vehicle technique for practical structural condition assessment of beam-like bridge deck. Remote Sens 12(1):114

[17] Mei Q, Shirzad-Ghaleroudkhani N, Gül M, Ghahari SF, Taciroglu E (2021) Bridge mode shape identification using moving vehicles at traffic speeds through non-parametric sparse matrix completion. Struct Control Health Monit 28(7):2747

[18] Corbally R, Malekjafarian A (2022) Bridge damage detection using operating deflection shape ratios obtained from a passing vehicle. J Sound Vib 537:117225

[19] Yang Y, Shi K, Wang Z, Xu H, Zhang B, Wu Y (2021) Using a single-dof test vehicle to simultaneously retrieve the first few frequencies and damping ratios of the bridge. Int J Struct Stab Dyn 21(08):2150108

[20] Li Z, Lin W, Zhang Y (2023) Real-time drive-by bridge damage detection using deep auto-encoder. In: Structures, vol 47. Elsevier, pp 1167–1181

[21] Corbally R, Malekjafarian A (2024) A deep-learning framework for classifying the type, location, and severity of bridge damage using drive-by measurements. Comput Aided Civ Infrastruct Eng 39(6):852–871

[22] Yang Y, Yang JP (2018) State-of-the-art review on modal identification and damage detection of bridges by moving test vehicles. Int J Struct Stab Dyn 18(02):1850025

[23] Malekjafarian A, Corbally R, Gong W (2022) A review of mobile sensing of bridges using moving vehicles: progress to date, challenges and future trends. In: Structures, vol 44. Elsevier, pp 1466–1489

[24] Mei Q, Gül M, Shirzad-Ghaleroudkhani N (2020) Towards smart cities: crowdsensing-based monitoring of transportation infrastructure using in-traffic vehicles. J Civ Struct Health Monit 10(4):653–665

[25] Matarazzo TJ, Kondor D, Milardo S, Eshkevari SS, Santi P, Pakzad SN, Buehler MJ, Ratti C (2022) Crowdsourcing bridge dynamic monitoring with smartphone vehicle trips. Commun Eng 1(1):29

[26] Kong X, Cai C, Kong B (2016) Numerically extracting bridge modal properties from dynamic responses of moving vehicles. J Eng Mech 142(6):04016025

[27] Eshkevari SS, Matarazzo TJ, Pakzad SN (2020) Bridge modal identification using acceleration measurements within moving vehicles. Mech Syst Signal Process 141:106733

[28] Quqa S, Giordano PF, Limongelli MP (2022) Shared micromobility-driven modal identification of urban bridges. Autom Constr 134:104048. 10.1016/j.autcon.2021.104048

[29] Yang Y-B, Chen W-F, Yu H-W, Chan C (2013) Experimental study of a hand-drawn cart for measuring the bridge frequencies. Eng Struct 57:222–231. 10.1016/j.engstruct.2013.09.007

[30] Li Z, Lan Y, Lin W (2023) Using contact residual responses of a 3-dof scooter to identify first few frequencies of the footbridge. In: International conference on experimental vibration analysis for civil engineering structures. Springer, pp 132–143

[31] Li Z, Lan Y, Lin W (2024) Indirect frequency identification of footbridges with pedestrians using the contact-point response of shared scooters. J Bridge Eng 29(6):04024036

[32] Gkoumas K, Gkoktsi K, Bono F, Galassi MC, Tirelli D (2021) The way forward for indirect structural health monitoring (ishm) using connected and automated vehicles in Europe. Infrastructures 6(3):43. 10.3390/infrastructures6030043

[33] Yang Y, Cheng M, Chang K (2013) Frequency variation in vehicle-bridge interaction systems. Int J Struct Stab Dyn 13(02):1350019. 10.1142/S0219455413500193

[34] Cantero D, McGetrick P, Kim C-W, OBrien E (2019) Experimental monitoring of bridge frequency evolution during the passage of vehicles with different suspension properties. Eng Struct 187:209–219. 10.1016/j.engstruct.2019.02.065

[35] Zhang Y, Wang L, Xiang Z (2012) Damage detection by mode shape squares extracted from a passing vehicle. J Sound Vib 331(2):291–307. 10.1016/j.jsv.2011.09.004

[36] Yang Y-B, Yang JP, Wu Y, Zhang B (2019) Vehicle scanning method for bridges. Wiley, Hoboken

[37] Corbally R, Malekjafarian A (2022) A data-driven approach for drive-by damage detection in bridges considering the influence of temperature change. Eng Struct 253:113783. 10.1016/j.engstruct.2021.113783

[38] Limebeer DJ, Sharp RS (2006) Single-track vehicle modeling and control. In: IEEE control systems magazine, pp 34–61

[39] Whipple FJ (1899) The stability of the motion of a bicycle. Q J Pure Appl Math 30(120):312–348

[40] Caprani CC, Ahmadi E (2016) Formulation of human-structure interaction system models for vertical vibration. J Sound Vib 377:346–367. 10.1016/j.jsv.2016.05.015

[41] Zhang M, Georgakis CT, Qu W, Chen J (2015) Smd model parameters of pedestrians for vertical human-structure interaction. In: Dynamics of civil structures, volume 2: proceedings of the 33rd IMAC, A conference and exposition on structural dynamics. 10.1007/978-3-319-15248-6_33. Springer, pp 311–317

[42] Polanco A, Marconi E, Muñoz L, Suárez D, Doria A (2019) Effect of rider posture on bicycle comfort. In: International design engineering technical conferences and computers and information in engineering conference, vol 59216. 10.1115/DETC2019-97763. American Society of Mechanical Engineers, pp 003–01025

[43] Viellehner J, Potthast W (2022) The effect of vibration on kinematics and muscle activation during cycling. J Sports Sci 40(15):1760–1771. 10.1080/02640414.2022.2109841 35984289 10.1080/02640414.2022.2109841

[44] OpenStreetMap. Copyright OpenStreetMap contributors. Data is available under the Open Database License. http://openstreetmap.org/copyright

[45] Hua Y, Sarkar TK (1990) Matrix pencil method for estimating parameters of exponentially damped/undamped sinusoids in noise. IEEE Trans Acoust Speech Signal Process 38(5):814–824. 10.1109/29.56027

[46] Magalhães F, Cunha A, Caetano E (2009) Online automatic identification of the modal parameters of a long span arch bridge. Mech Syst Signal Process 23(2):316–329. 10.1016/j.ymssp.2008.05.003

[47] Cheynet E, Jakobsen JB, Snæbjörnsson J (2016) Buffeting response of a suspension bridge in complex terrain. Eng Struct 128:474–487. 10.1016/j.engstruct.2016.09.060

[48] Cheynet E (2020) Operational modal analysis with automated SSI-COV algorithm. Zenodo. 10.5281/ZENODO.3774061

[49] Welch P (1967) The use of fast Fourier transform for the estimation of power spectra: a method based on time averaging over short, modified periodograms. IEEE Trans Audio Electroacoust 15(2):70–73. 10.1109/TAU.1967.1161901

[50] ...Virtanen P, Gommers R, Oliphant TE, Haberland M, Reddy T, Cournapeau D, Burovski E, Peterson P, Weckesser W, Bright J, van der Walt SJ, Brett M, Wilson J, Millman KJ, Mayorov N, Nelson ARJ, Jones E, Kern R, Larson E, Carey CJ, Polat İ, Feng Y, Moore EW, VanderPlas J, Laxalde D, Perktold J, Cimrman R, Henriksen I, Quintero EA, Harris CR, Archibald AM, Ribeiro AH, Pedregosa F, van Mulbregt P (2020) SciPy 1.0 contributors: SciPy 1.0: fundamental algorithms for scientific computing in Python. Nat Methods 17:261–272. 10.1038/s41592-019-0686-2 32015543 10.1038/s41592-019-0686-2 PMC7056644

[51] Scipy.signal documentation. Estimate power spectral density using Welch’s method. https://docs.scipy.org/doc/scipy/reference/generated/scipy.signal.welch.html. Accessed 23 Dec 2022

[52] Visual Crossing. Weather Data & API. https://www.visualcrossing.com/

[53] Jazar RN (2008) Vehicle dynamics, vol 1. Springer, New York

[54] SciPy documentation. Short Time FFT. https://docs.scipy.org/doc/scipy/reference/generated/scipy.signal.ShortTimeFFT.html. Accessed 29 June 2024

[55] Wynne Z (2022) Closing the loop: the integration of long-term monitoring in engineering design practice. Ph. D. thesis, The University of Edinburgh. 10.7488/era/2694

[56] May R, Chai HK, Reynolds T, Lu Y (2023) Exploring the use of bicycles as exciters and sensor carriers for indirect bridge modal parameter estimation. In: International conference on experimental vibration analysis for civil engineering structures. 10.1007/978-3-031-39117-0_26. Springer, pp 254–263

